# Neuropathic Itch

**DOI:** 10.3390/cells9102263

**Published:** 2020-10-09

**Authors:** James Meixiong, Xinzhong Dong, Hao-Jui Weng

**Affiliations:** 1 Solomon H. Snyder Department of Neuroscience and Medical Scientist Training Program, Johns Hopkins University School of Medicine, Baltimore, MD 21205, USA; jmeixio1@jhmi.edu; 2 Solomon H. Snyder Department of Neuroscience, Department of Dermatology, and Department of Neurosurgery, Johns Hopkins University School of Medicine, Baltimore, MD 21205, USA; xdong2@jhmi.edu; 3 Howard Hughes Medical Institute, Johns Hopkins University School of Medicine, Baltimore, MD 21205, USA; 4 Department of Dermatology, Taipei Medical University-Shuang Ho Hospital, New Taipei City 23561, Taiwan; 5 Department of Dermatology, School of Medicine, College of Medicine, Taipei Medical University, Taipei 11031, Taiwan

**Keywords:** pruritus, neuropathy, inflammation

## Abstract

Neurologic insults as varied as inflammation, stroke, and fibromyalgia elicit neuropathic pain and itch. Noxious sensation results when aberrantly increased afferent signaling reaches percept-forming cortical neurons and can occur due to increased sensory signaling, decreased inhibitory signaling, or a combination of both processes. To treat these symptoms, detailed knowledge of sensory transmission, from innervated end organ to cortex, is required. Molecular, genetic, and behavioral dissection of itch in animals and patients has improved understanding of the receptors, cells, and circuits involved. In this review, we will discuss neuropathic itch with a focus on the itch-specific circuit.

## 1. Introduction

Both microscopic damage, such as small-fiber neuropathy, and macroscopic trauma such as that occurring from spinal cord transection can result in neuropathic pain or itch [1]. Neuropathic pain is defined by the International Association for the Study of Pain (IASP) as pain caused by a lesion or disease of the somatosensory nervous system [2]. Currently, neuropathic itch has no consensus definition but is generally understood as itch resulting from neuronal or glial damage without skin alterations. Under this definition, neuropathic itch accounts for approximately 8% to 19% of chronic pruritus conditions and include a wide variety of neurologic diseases with high medical burden and diverse pathologic mechanisms [3].

Pain- and itch-detecting neurons are anatomically indistinguishable. Both are sensed by small-diameter, unmyelinated C-fibers of the dorsal root ganglia (DRG) and trigeminal ganglia (TG). These unipolar primary neurons synapse in the outer layers of the dorsal horn of the spinal cord. Signal-carrying, secondary spinal neurons anteriorly decussate before traveling rostrally in the spinothalamic and spinobrachial tracts [4]. In human psychophysical studies, participants often report coexisting pain and itch sensations (“stinging itch” and “itching burn”) in response to itchy and painful exogenous stimuli. Intriguingly, after nerve injury, sufferers commonly complain of concurrent pain and itch [1,5,6,7]. For example, 92% of patients with small fiber neuropathies experience coexisting neuropathic itch and pain [8]. Similarly, after herpes zoster virus reactivation, 30–65% of patients experience both neuralgia and itch as a result of their illness [6,7].

In addition to widespread musculoskeletal-type pain and systemic symptoms of altered mood, fatigue, and cognitive dysfunction, patients with fibromyalgia have exquisitely sensitized skin [9,10]. A subset of patients with fibromyalgia have skin-related complaints including hyperhidrosis, burning pain, and unexplained pruritus [11,12]. However, reported estimates of prevalence widely vary, 3.4–52% for cutaneous pain and 3.3–73% for pruritus [11,12]. On skin biopsy, approximately 40% of fibromyalgia patients have evidence of small-fiber polyneuropathy suggestive of a neuropathic cause for their altered sensation.

In recent years, advances in molecular biology have identified itch-specific sensory neurons and allowed detailed dissection of both pain and itch circuits. From these studies, a comprehensive framework of noxious sensation, from molecule to cell to circuit and perception, has emerged. Although itch neurons are molecularly distinct from their pain-sensing counterparts, neuropathic itch shares common mechanisms with neuropathic pain. Understanding how nervous damage and disease can result in pathologic itch can allow better explanation and management of the pruritus and cutaneous symptoms experienced by fibromyalgia patients. In this review, we will discuss recent advances in itch biology, the itch-specific circuit, and pathophysiologic mechanisms of neuropathic itch.

## 2. Neuropathic Itch and Inflammation

Itch sensation begins in the skin when varied pruritogenic stimuli: molecules, temperature, and mechanical force, excite afferent nerves through a variety of cell surface receptors and channels. Receptors and channels confer molecular specificity, and their expression dictates if, when, where, and how an itch neuron is excited. Single-cell sequencing studies have provided a near-complete account of receptors and channels expressed in itch and pain-sensing neurons during various dermatoses, so-called “itchscriptomes,” expanding our molecular understanding of itch far beyond the canonical histamine receptor implicated in allergic itch [13,14,15,16,17].

**Figure 1:**
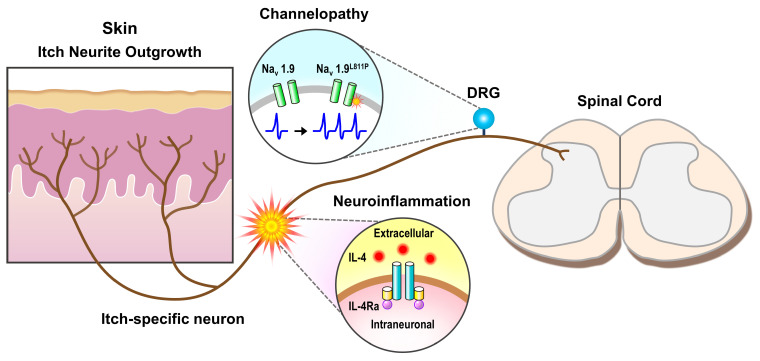
Neuropathic itch of the peripheral nervous system. Small diameter itch-sensing nerves of the dorsal root ganglia (DRG) ramify in skin and synapse with secondary neurons in the outer laminar layers of the dorsal horn of the spinal cord. Various insults along this peripheral neuronal tract can result in neuropathic itch. These include increased neurite outgrowth in diseased skin, neuroinflammatory activation via IL-4/IL-4Ra receptor, and genetic channelopathies such as Na_v_1.9^L811P^ which has been associated with increased itch nerve activity.

In allergic itch and numerous chronic itch conditions, a component of itch sensation can be attributed to a pathologic increase of pruritogen in skin which activates receptors and/or channels present on itch neurons. This mechanism, whereby extrinsic pruritogen increases itch afferent signaling, applies to neuropathic itch in certain clinical contexts. After peripheral nerve injury, significant inflammation concurrently develops with sensory abnormalities (Figure 1) [4,18]. Itch neurons express a variety of receptors for inflammatory mediators such as proteases, tryptase, chymase, and cathepsin, and cytokines, thymic stromal lymphopoietin, interleukin (IL)-4, 13, 31, and 33 [16,19,20,21,22,23]. Based on this receptor expression, it is plausible that a component of neuropathic itch is due to activation of receptors by inflammatory molecules. For example, IL-4 activation of IL-4Rα, an interaction found to be important in chronic itch of atopic dermatitis, could play a role in peripheral nerve injury-associated neuropathic itch. At days 1 and 7 following sciatic nerve transection, IL-4 is significantly upregulated in the distal nerve stump [4]. Both mouse and human itch neurons express IL-4Rα, the receptor for IL-4 [19].

Historically, when inflammatory skin changes prevail, such as in psoriasis, atopic dermatitis, or allergic contact dermatitis, associated itch has been classified as inflammatory and not neuropathic. However, data suggests that these processes may be linked. During inflammation, keratinocytes release neuro-active molecules such as nerve growth factor (NGF), amphiregulin, artemin, sempahorins, and anosmin-1 which alter nerve fiber growth and proliferation [24]. In mouse models of allergic contact dermatitis, inflamed skin exhibits nerve hyperproliferation and alters sensory neuron expression profiles. In several inflammatory dermatoses, including psoriasis, systemic sclerosis, morphea, dermatomyositis, and atopic dermatitis, neuropathic-type itch, pain, and other symptoms have been documented [24,25,26]. Inflammation increases intraepidermal nerve fiber density. However, in non-inflamed skin of patients complaining of neuropathic itch, intraepidermal nerve fiber density is often decreased [8]. This phenomenon reflects one potential pathogenic difference between inflammatory and neuropathic itch.

## 3. Neuropathic Itch and Channelopathies

In peripheral nerves, receptor activation often recruits downstream ion channels, causing influx of positive ions and neuronal excitation. However, channels themselves can be directly activated, independent from receptor recruitment, to excite afferent neurons and produce neuropathic itch sensation. For example, voltage-gated sodium channels (Na_v_), expressed in small-diameter sensory neurons of the DRG, TG, and sympathetic ganglia, have been linked to neuropathic itch. These channels regulate neuronal excitability and action potential propagation by altering resting membrane potential and inactivation thresholds. Numerous gain-of-function mutations that serve to increase neuronal excitability have been identified as causing neuropathic itch in patients [27,28,29,30,31]. For example, three patients with paroxysmal bouts of severe itch affecting the trunk and upper extremities were found to have abnormal sensory thresholds due to a Na_v_1.7^I739V^ mutation [29]. Similarly, an additional patient complaining of severe, unrelenting itch, was identified as harboring a mutation in a different channel, Na_v_1.9^L811P^ [31]. In the described patients, severe, neuropathic itch occurred in the absence of any known itch-associated pathology or even overt damage to the nervous system. All of the mentioned patient’s neuropathic itch responded well to gabapentin [29,31]. Differences in symptom chronicity between these patients, paroxysmal vs. constant, can be explained by the dynamics of the mutated sodium channel. Distinct from other voltage-gated sodium channels, Na_v_1.9 produces a distinct current at physiologically-relevant resting membrane potentials [30]. Alterations to this current could affect neuronal firing, even in the absence of external stimuli.

## 4. Cellular Mechanisms of Neuropathic Itch

**Figure 2:**
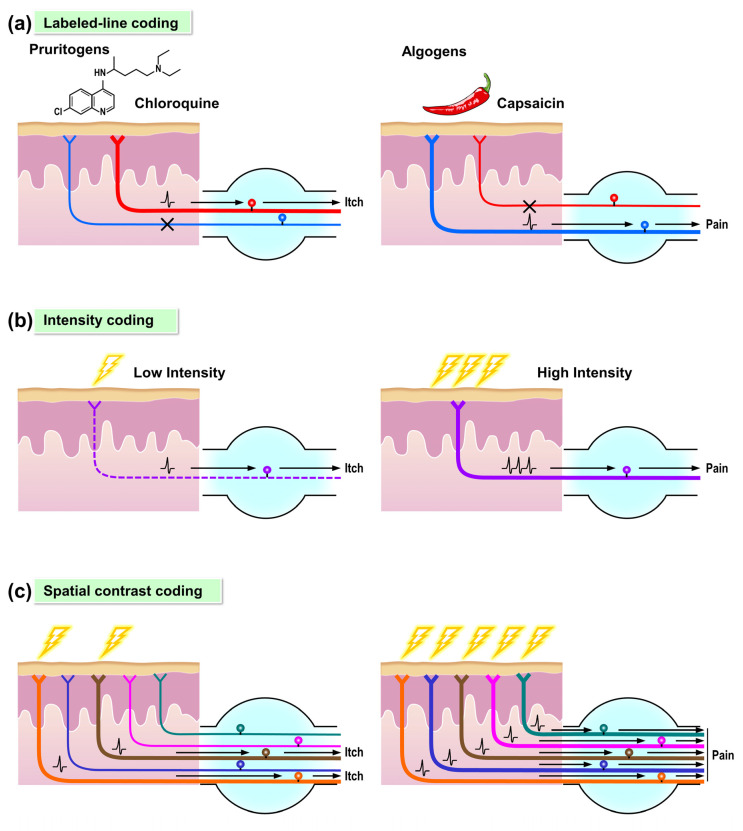
Models of coding for itch and pain. Different models of coding have been proposed to explain the relationship between itch and pain. (**a**) In the labeled-line coding model, the itch-specific sensory neuron (left) and pain-specific neuron (right) each constitutes a dedicated pathway that only responds to corresponding pruritogens (i.e., chloroquine) or algogens (i.e., capsaicin). (**b**) In the intensity coding model, the itch and pain pathways are comprised of the same population of sensory neurons and circuits. Low-to medium intensity stimuli evoke low firing rates of the sensory neurons and result in itch sensation (left). On the contrary, high-intensity stimuli evoke robust firing rates of the same sensory neurons and result in pain sensation (right). (**c**) In the spatial contrast coding model, focal stimulation of the individual nociceptors induces itch sensation (left). Contrarily, co-stimulation of the same population of sensory neurons with the neighboring nociceptors evokes the pain sensation (right).

In addition to molecular causes, there are numerous cell-based mechanisms by which peripheral lesions can increase afferent signaling and elicit neuropathic itch. To understand these, the way itch is coded in the periphery should be discussed. Although the mechanism of peripheral itch-coding is still debated, synthesis of molecular, physiologic, and human psychophysical data supports the existence of itch-specific sensory neurons, labeled-line coding, which may selectively innervate skin sites in support of the spatial contrast model (Figure 2a,c) [5].

### 4.1. Molecular Data Support a Labeled-Line for Itch 

Itch neurons are small-diameter, pseudounipolar cells that reside within the DRG and TG. They maintain a single axon that innervates both peripheral skin and synapses with secondary spinal cord neurons. Single-cell sequencing studies have demonstrated that itch neurons, classified based on canonical itch receptor expression, are molecularly distinct from other sensory neurons including those that detect pain [13]. Functional genetic studies enabling activation of specific neural populations in mice also support the existence of distinct, itch-coding neurons in both the peripheral and central nervous systems. In the periphery, natriuretic polypeptide B (NPPB) and Mrgpra3-expressing neurons are believed to represent itch-exclusive neuronal populations [32,33]. Exclusive, robust activation of Mrgpra3-expressing sensory neurons in mice elicits itch and not pain [32]. NPPB is both necessary and sufficient for acute peripheral itch. Injection of NPPB produces exclusive itch, and NPPB^−/−^ mice have specific itch deficits with intact pain [32,33]. In the outer lamina of the dorsal horn of the spinal cord, peripheral neurons synapse with itch-dedicated secondary neurons expressing NPRA, the NPPB receptor, and gastrin-releasing peptide (GRP). Secondary neurons transmit a signal to tertiary, GRP receptor-expressing (GRPR^+^) pruriceptors [33,34,35]. GRPR^+^ neuron ablation decreases itch without altering pain, and GRP injection elicits exclusive itch.

### 4.2. Alternative Itch Coding Mechanisms

In human psychophysical studies where pruritogens are injected intradermally, mixed pain, and itch sensations, a “stinging itch” or “itching burn,” are commonly reported [36,37,38,39]. Canonical pruritogens such as histamine and cowhage induce stinging and burning pain in some volunteers, while stereotypic algogens such as capsaicin can provoke itch [38,39]. Concurrently, electrophysiologic recordings from a variety of mammalian models demonstrate that pruriceptive neurons also respond to painful stimuli [40,41]. In murine spinal cords, evidence that GRP^+^ neurons represent a population of neurons that code for both pain and itch has been presented [42]. Specific, low-to-medium intensity activation of these neurons elicited pain and itch, and maximal activation elicited exclusive itch, a pattern of activity consistent with a type I incoherent feedback circuit [42].

Labeled-line coding cannot completely account for these observed data, which are more accurately described by alternative theories of itch signaling, intensity, and spatial contrast coding [5]. Under these paradigms, itch signaling is decoded by physiologic parameters and not necessarily coded by distinct neural circuits. Intensity coding proposes that a single population of nociceptors detect itch and pain. In this model, low firing rates result in itch and robust firing rates are decoded as pain (Figure 2b). Alternatively, in spatial contrast coding, the regional context of nociceptor activation matters (Figure 2c). For example, focal nociceptor stimulation would be expected to induce itch. From these stimuli, contrasted signaling from a few, active nociceptive endings and surrounding, silent nociceptors codes for itch. Specific, in vivo testing of spatial contrast coding has yet to be performed. However, in mice, particularly strong evidence against intensity coding exists as maximal activation of specific sensory neuron populations, such as Mrgpra3^+^ neurons in mice, elicits exclusive itch without transition to pain [32,43,44].

## 5. Neuropathic Itch of Peripheral Nerve Neuromas

As no single itch-coding theory accounts for all experimental data, peripheral itch-coding in humans likely involves a combination of mechanisms. Synthesis of existing theory best explains the cellular mechanisms by which peripheral neural lesions cause neuropathic itch. For example, after limb amputation or traumatic surgical procedures such as mastectomy, itch of the deafferented region, so-called “phantom” itch, commonly occurs [3]. During wound healing, substantial cellular reorganizations of the peripheral nervous system, such as neuromas, increased neurite outgrowth and nerve embedment in scar tissue can develop [3,45]. Similar increases in small-fiber, nociceptive innervation are observed in normal and hypertrophic keloid scar tissue, skin graft, and post-burn injury, all conditions associated with significant itch incidence [46,47,48,49]. One explanation for itch in this context would be that the assayed, increased innervation exclusively represents labeled-line itch fiber proliferation. However, these straightforward explanations are less likely as neuropeptide and neural marker staining of scar support the presence of both pain and itch neurons [47,49].

As in other neuropathic conditions, patients with phantom limb sensation commonly report concurrent itch and pain but can also experience isolated pain or itch [50]. An admixture of sensations is best explained by a combination of spatial contrast and labeled-line signaling. In the context of a neuroma and resultant nerve ending hyperproliferation, spatial contrast theory can account for isolated itch, isolated pain, and the switch between these sensory states. When there is no spatial contrast in neuronal firing in the hyper-innervated injury site, such as in an inflamed neuroma where nerve endings are globally activated, pain results. When different regions of the neuroma are activated, such as in subtle inflammation or due to focal, external stimuli, spatial contrast is present, and itch is sensed. How concurrent itch and pain sensation can be detected is more difficult to explain by spatial contrast coding. Neuromas at amputation sites, hypertrophic scar, and burn scar have denser neuronal innervation compared to normal skin. In this anatomically crowded space, achievement of spatial contrast would require extraordinarily specific activation [51].

Labeled-line coding can resolve this issue of mixed pain and itch sensation. In a peripheral neuroma, a subset of proliferating nerve endings will be itch-specific and others, pain-specific. When all are activated, such as in inflammation, both pain and itch sensation can be detected. Labeled-line coding could theoretically account for exclusive pain and exclusive itch sensations resulting from a neuroma even when both pain and itch-specific afferents are activated. However, each explanation assumes much. Pain inhibits itch in the spinal cord, a topic which will be explored further in the next section. In an inflamed neuroma, exclusive pain would result if pain inhibits itch sensation. Exclusive itch resulting from a neuroma is more difficult to account for with labeled-line coding. After peripheral nerve transection injury, sensory neurons of the dorsal root ganglia undergo significant molecular changes, including broad upregulation of the itch neuropeptide GRP, which could abrogate typical nociceptor inhibition of itch through volume transmission [52]. However, for exclusive itch to be detected in this scenario, injury-related changes would have to result in near-complete sensory neuron expression of GRP which has not been experimentally demonstrated. Alternatively, exclusive itch could be sensed if stimuli were applied with molecular specificity, activating only itch and not pain neurons. However, in humans, few physiologic molecules that elicit pure itch have been identified. With these caveats in mind, a synthesized mechanism of both labeled-line and spatial contrast coding would best explain the neuropathic sensations evident in phantom limbs.

## 6. Circuit Mechanisms of Neuropathic Itch in the Spinal Cord

**Figure 3:**
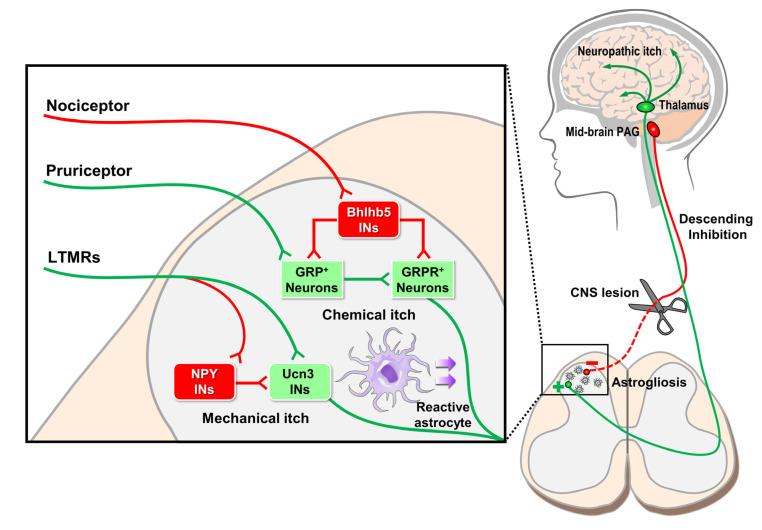
Neuropathic itch of the central nervous system. Peripheral itch nerves synapse with secondary Gastrin-releasing peptide (GRP)^+^ itch neurons of the spinal cord which then activate tertiary GRP receptor (GRPR)^+^ neurons. GRPR^+^ neurons decussate anteriorly to form the spinothalamic tract and synapse with thalamic neurons which communicate with various brain regions such as the sensory cortex and insula to produce itch sensation. In the spinal cord, numerous interneuron (INs) populations control itch processing. For example, nociceptor activation of Bhlhb5 INs inhibits GRP^+^ and GRPR^+^ neuron signaling of chemical itch. Similarly, peripheral low-threshold mechanoreceptors (LTMRs) can both enhance mechanical itch by activating excitatory Ucn3 INs and inhibit mechanical itch via inhibitory NPY INs. In addition to these peripheral inputs, higher-level brain regions, such as the mid-brain periaqueductal gray (PAG), provide descending inhibitory feedback onto spinal cord itch neurons. If descending inhibitory pathways are disrupted, then itch becomes disinhibited, and neuropathic itch results. Finally, chronic itch conditions can cause reactive spinal astrogliosis, a non-neuronal mechanism whereby itch signaling is enhanced.

Peripheral nerve damage affects not only primary DRG neuron gene expression, such as GRP upregulation but also that of non-neuronal cell types in the spinal cord such as astrocytes and microglia (Figure 3). During peripheral nerve injury, spinal cord astrocytes and microglia undergo reactive gliosis, a process that includes proliferation, upregulation of cytokine production and release, and morphologic changes [53,54,55]. Typically, spinal microglial plasticity is transitory and peaks within days, while astrocytic changes persist for weeks to months [56,57].

Both microglial and astrocytic changes have been shown to play a role in neuropathic pain. For example, after nerve injury, both cell types release pro-inflammatory cytokines and chemokines, such as CCL2 and CXCL1, which sensitize pain-coding spinal cord neurons and make them more responsive to peripheral stimuli [56]. In neuropathic itch, a similar mechanism whereby gliosis promotes itch sensation by modulating itch circuits may be present. In mice with chronic itch of atopic and contact dermatitis, reactive astrogliosis in the spinal cord is both critical for the development of itch and also enhances the sensation through release of lipocalin-2, which sensitizes GRPR^+^ neurons to GRP itch neuropeptide transmission [58,59]. Similar dynamic glial changes could account for neuropathic pruritus observed in neuromyelitis optica spectrum disorders (NOSD), a neuroinflammatory disorder with auto-antibody targeting aquaporin-4 leading to demyelination and astrogliosis of the spinal cord [60].

In addition to glia, inhibitory and excitatory spinal interneurons of the dorsal horn also regulate itch transmission (Figure 3). Two separate inhibitory interneuron populations, basic helix-loop-helix b5 (Bhlhb5)-dependent and neuropeptide Y (NPY)^+^ inhibitory interneurons link pain and mechanosensation, respectively, to itch signaling [61,62]. Bhlhb5-dependent interneurons gate chemical itch while NPY^+^ interneurons gate mechanical itch. In the circuits formed, pain and mechanosensory afferent signaling activates respective interneuron populations which inhibit itch. Loss of either interneuron population results in severe itch in the absence of peripheral stimuli without altering other sensations [61,62]. Excitatory spinal interneurons have also been shown to modulate itch. In mechanical itch, urocortin 3- expressing excitatory interneurons are critical for mechanical itch and mechanical alloknesis in chronic itch conditions [63].

Understanding spinal cord itch regulation sheds light on the pathogenesis of neuropathic itch conditions such as trigeminal trophic syndrome (TTS). TTS is a severe neuropathic itch that results after cutaneous pain neuron deafferentation due to shingles, treatments of trigeminal neuralgia such as rhizotomy and ethanol injection, and vertebrobasilar stroke affecting the trigeminal nucleus [64]. Because pain afferent signaling inhibits itch in the spinal cord, when pain afferents are lesioned via surgical intervention or viral infection, itch is disinhibited and the responses are more pronounced when activated. TTS patients will scratch themselves until skin ulcerates [64]. Because patients feel itch and no pain, they have the motivation to scratch and no awareness of the extent of self-inflicted damage caused. Similarly, in mice, silencing of peripheral pain by targeting TRPV1^+^ afferent signaling also elicits severe, pathologic itch [65].

While the absence of pain and resultant disinhibition of itch can account for the severity of itch perceived by TTS patients, it cannot explain why itch occurs in the first place and appears to be such an entrenched phenotype. Peripheral itch afferents are not tonically active. Even in the complete absence of pain and spinal inhibitory neuron input, the itch neural circuit would still require peripheral stimuli to become active.

Reactive astrogliosis could account for tonic itch sensation and repetitive scratching in the absence of peripheral stimuli by promoting the development of an “itch-scratch cycle.” Itch-scratch cycle is a positive feedback loop that develops in nearly all chronic itch conditions where scratch enhances rather than attenuates itch. Scratch activates TRPV1^+^ peripheral afferents which promote reactive astrogliosis, release of factors such as LCN2, and sensitization of itch neurons. Astrogliosis is a persistent phenomenon that, once developed, lasts for many months, a timescale wherein significant tissue damage such as that seen in TTS can occur.

## 7. Descending Supraspinal Control of Itch Sensation

In the spinal cord, itch sensation is modulated not only by local segmental neurons and glia but also by distant supraspinal neurons from varying regions such as the dorsal reticular nucleus, mid-brain periaqueductal gray-rostral ventromedial medulla (RVM), and ventrolateral medulla (VLM). Descending control is state-dependent [66,67]. During stress-associated fight-or-flight response, descending control is overall inhibitory and produces hypoalgesia, and in the context of diseases such as peripheral nerve injury, descending input facilitates pain by enhancing pain transmission in the spinal cord [66,68].

While descending control can facilitate or inhibit sensation in a context-dependent manner, overall it is thought to be inhibitory. When supraspinal control centers are experimentally lesioned, sensation is increased, presumably due to loss of descending inhibitory control and subsequent disinhibition [66]. Clinical evidence also supports an overall inhibitory role for descending control pathways. Lesions to supraspinal itch control centers such as the lateral medulla in Wallenberg’s syndrome can result in neuropathic itch. Similarly, destruction of descending fibers in the spinal cord due to tumor, trauma, multiple sclerosis, or infarct, or stroke are also associated with severe neuropathic itch [1,69]. In chronic itch disease of atopic dermatitis, prurigo nodularis, and neuropathic brachioradial pruritus, descending control is altered. Typically, in healthy people, repeated pain stimulation results in decreased pain ratings due to centrally-mediated desensitization [53]. However, in chronic itch patients, this desensitization is not observed, indicating that descending inhibitory pathways are lost [70].

Faciliatory and inhibitory descending control fibers are intermixed, and their cell bodies are closely associated anatomically. For example, within the ventrolateral periaqueductal gray (vlPAG), a midbrain structure just a few millimeters in size, both inhibitory and faciliatory cell types for descending control of itch exist. While pharmacologic inhibition of the entire structure attenuates itch, suggesting that the vlPAG as a whole facilitates itch, cell-type-specific activation of glutamatergic neurons inhibits itch while GABAergic neurons facilitate itch [71]. Given the presence of both faciliatory and inhibitory descending control of sensation, it is curious why neuropathic itch and pain are more commonly reported in association with spinal cord lesions than the corollary loss of sensation. Such differences may be due to reporting bias where patients who are experiencing noxious percepts are more likely to bring these symptoms to a medical provider’s attention.

In addition to discrete lesions of the spinal cord and brainstem, diffuse cortical disease, such as in Lewy body dementia and Crueztfeldt–Jakob prion disease (CJD), has also been associated with increased itch sensation [72,73]. After synapsing in thalamus, itch sensation projects to basal ganglia and many parts of the cortex including the somatosensory cortex, premotor cortex, the posterior parietal cortex, anterior cingulate, and insula. Different itch stimuli preferentially activate specific cortical regions. For example, allergen-associated itch activated supplementary motor cortex, an activity which was not apparent in histamine-associated itch [74].

One mechanism by which aberrant itch could develop in global cortical neurodegenerative conditions would be by disruption of descending inhibitory control neurons similar to that previously discussed with discrete lesions. Parietal stroke can cause neuropathic itch, and in familial, but not sporadic CJD, diffusion-weighted imaging revealed decreased activity in mid-brain periaqueductal gray, suggesting possible alterations to inhibitory pathways located there ultimately resulting in increased itch [75,76]. However, these affected patients had severe disease, and many other itch-associated CNS regions were similarly affected [75].

Psychogenic itch such as delusional parasitosis in Ekbom’s and Morgellon’s syndromes is a relatively uncommon but notable cause of chronic itch [77,78]. The neural correlates for these conditions are still under active investigation and could involve unidentified disruption of descending itch control pathways in addition to psychiatric causes. Intriguingly, cerebral infarct of the right medial occipital lobe can elicit delusional parasitosis, indicating that a single, discrete lesion, disrupting select neural pathways, can explain disease symptoms [79].

Mechanistic studies of psychogenic itch are challenging due to the close relationship itch has with psychiatric conditions. Chronic itch leads to depression and anxiety, which in turn can enhance sensation of itch [80]. This mixed causality makes it difficult to distinguish primary drivers of disease. However, peripheral itch was once just as mysterious as more central causes. By correlating clinical findings of patient complaint with itch sensory research, enormous strides have been made in understanding the peripheral and spinal circuits of itch. Future studies on itch processing in higher-level brain regions can be similarly conducted and provide knowledge which will benefit those who are suffering.

## 8. Future Therapeutic Directions for Neuropathic Itch

Understanding the molecular, cellular, and circuit regulation of itch sensation provides opportunities for improved therapeutics [81,82,83]. In recent years, treatment of inflammatory itch associated with atopic dermatitis, psoriasis, and prurigo nodularis has been revolutionized by identification of inflammatory molecules and receptors involved [19]. As neuropathic itch can develop following neuroinflammatory injury, it is possible that some of the therapeutics shown to be helpful for atopic dermatitis might also benefit a subset of neuropathic itch patients. Once limited to barrier treatment, steroid creams, and anti-histamines, improved, specific treatments targeting cytokine mediators such as IL-4/IL-13 (dupilumab), IL-17 (secukinumab and ixekizumab), IL-31RA (nemolizumab), and JAK-STAT receptors have greatly improved patient outcomes [19,84,85,86,87,88]. Another drug class which has shown promise is neurokinin 1 (NK1) receptor antagonists. In multiple phase II trials, the NK1 antagonist serlopitant showed significant anti-itch effects in patients with prurigo nodularis and chronic itch conditions [89,90]. Unfortunately, neither trial tested the therapeutic effect of serlopitant for neuropathic itch [89,90].

Neuropathic itch treatments being actively explored include injections of botulinum toxin and topical applications of ketamine mixtures or strontium. Botulinum toxin has been shown to improve, although with mixed results, neuropathic itch in brachioradial pruritus, notalgia paresthetica, and keloids [91,92,93,94]. Topical application of ketamine, mixed with amitryptyline and/or lidocaine, provided immediate relief for neuropathic itch patients [95]. Finally, topical application of strontium, a calcimimetic calcium channel blocker, has benefited experimentally induced histaminergic and non-histaminergic itch and, theoretically, could relieve neuropathic itch [96]. Depending on context, symptomatic neuropathic itch and pain have been treated primarily by therapies which similarly aim to decrease afferent signaling, whether by inhibition of excitatory ion channels with lidocaine, activation of inhibitory ion channels with gabapentin, or ablation of afferent neurons with high dose capsaicin. Further study determining the cytokines, chemokines, and receptors involved in neuroinflammatory injury would be of particular therapeutic interest.

Understanding the circuit mechanisms of itch regulation provides additional opportunities for therapeutic intervention upon neuropathic itch. Based on current circuit models, to decrease itch, one could increase pain afferent signaling, increase local spinal cord inhibitory interneuron signaling, or increase descending inhibitory signaling. Certain therapeutic routes are clinically untenable, for example, treating itch by provoking pain. However, other options, such as increasing local inhibitory interneuron signaling, remain viable. To increase spinal cord inhibitory neuron signaling, one could either specifically activate those populations of neurons or introduce selective neurotransmitters, if they exist, released by those neurons. Pain-activated spinal interneurons inhibit itch through the release of dynorphin, a kappa opioid receptor agonist, suggesting that this class of molecules could treat itch conditions [97]. Indeed, kappa opioid agonists such as nalfurafine and difelikefalin have shown clinical promise for treatment of a variety of chronic pruritic conditions and similar treatments could also benefit neuropathic itch associated with peripheral nerve injury [98].

## 9. Conclusions

Neuropathic itch can result from almost any process that damages the nervous system. Many types of damage directed at a myriad of anatomic locations are associated with neuropathic itch. For this symptom to be controlled in the population at large, multiple therapeutic interventions, operating through separate mechanisms, are required. Increased understanding of itch transmission, from molecule to circuit and from skin to cortex, has similarly improved knowledge of neuropathic itch pathophysiology. As data concerning the molecules, cells, and circuits of itch continue to be refined, treatments for neuropathic itch will improve.

Despite the recent scientific focus on the distinction between itch and pain, their commonalities, in terms of molecules, cells, and circuits, are undeniable. Pain and itch are elicited by similar stimuli, chemical, temperature, and mechanical. While, overall, they are molecularly distinct, both types of neurons shared expression of key channels and receptors such as TRP channels. Both sensations are carried by similar size neurons that travel along the same neuroanatomical tracts to be processed in the same brain regions. Similar logic is employed in their control, whether by distant descending control pathways or local spinal interneuron circuits. For example, in an analogous mechanism to itch inhibition by pain, pain itself is gated by touch sensation. Based on these similarities, the scientific principles upon which successful neuropathic itch treatments are based will benefit pain just as knowledge of neuropathic pain has long informed efforts to understand itch.
